# Differences in Exercise Capacity, Ventilatory Efficiency, and Gas Exchange between Patients with Pulmonary Arterial Hypertension and Chronic Thromboembolic Pulmonary Hypertension Residing at High Altitude

**DOI:** 10.31083/j.rcm2507247

**Published:** 2024-07-04

**Authors:** Mauricio Gonzalez-Garcia, Rafael Conde-Camacho, Katherine Díaz, Camilo Rodríguez-Cortes, Emily Rincon-Alvarez

**Affiliations:** ^1^Fundación Neumológica Colombiana, 110131 Bogotá, Colombia; ^2^Faculty of Medicine, Universidad de la Sabana, 250001 Chía, Colombia; ^3^Postgraduate Program in Sports Medicine, Universidad El Bosque, 110121 Bogotá, Colombia

**Keywords:** pulmonary arterial hypertension, chronic thromboembolic pulmonary hypertension, altitude, exercise tolerance, cardiopulmonary exercise test, blood gas analysis

## Abstract

**Background:**

Cardiopulmonary exercise testing (CPET) assesses exercise capacity and causes of exercise limitation in patients with pulmonary hypertension (PH). At altitude, changes occur in the ventilatory pattern and a decrease in arterial oxygen pressure in healthy; these changes are increased in patients with cardiopulmonary disease. Our objective was to compare the response to exercise and gas exchange between patients with pulmonary arterial hypertension (PAH) and chronic thromboembolic pulmonary hypertension (CTEPH) residing at the altitude of Bogotá (2640 m).

**Methods:**

All patients performed an incremental CPET with measurement of oxygen consumption (${\mathrm{VO}}_{2}$), dead space (VD/VT), ventilatory equivalents (VE/${\mathrm{VCO}}_{2}$), and alveolar–arterial oxygen gradient (${\mathrm{PA-aO}}_{2}$). $X^{2}$ test and one-way analysis of variance were used for comparisons between PAH and CTEPH.

**Results:**

We included 53 patients, 29 with PAH, 24 with CTEPH, and 102 controls as a reference of the normal response to exercise at altitude. CTEPH patients had a higher New York Health Association (NYHA) functional class than PAH (*p* = 0.037). There were no differences between patients with PAH and CTEPH in hemodynamics and ${\mathrm{VO}}_{2}$% of predicted (67.8 ± 18.7 vs. 66.0 ± 19.8, *p* < 0.05), but those with CTEPH had higher dyspnea, VD/VT (0.36 ± 0.09 vs. 0.23 ± 0.9, *p* < 0.001), VE/${\mathrm{VCO}}_{2}$ (45.8 ± 7.1 vs. 39.3 ± 5.6, *p* < 0.001), and ${\mathrm{PA-aO}}_{2}$ (19.9 ± 7.6 vs. 13.5 ± 7.6, *p* < 0.001) than PAH patients.

**Conclusions:**

At altitude, patients with PH present severe alterations in gas exchange during exercise. There were no differences in exercise capacity between PAH and CTEPH, but patients with CTEPH had more dyspnea and greater alterations in gas exchange during exercise. CPET made it possible to identify alterations related to the pathophysiology of CTEPH that could explain the functional class and dyspnea in these patients.

## 1. Introduction

Pulmonary hypertension (PH) is a chronic and progressive disease that increases pulmonary arterial pressure (PAP) and pulmonary vascular resistance (PVR), ultimately leading to right ventricular failure. Regardless of the underlying disease, PH is almost always associated with progressive exercise intolerance, dyspnea, and increased mortality [1, 2, 3].

In clinical settings, the cardiopulmonary exercise test (CPET) is very useful for pulmonologists and cardiologists in the follow-up of patients with PH. It is used to evaluate exercise tolerance, exertional dyspnea, and related underlying pathophysiological mechanisms; it can suggest the diagnosis of PH and differentiate between possible causes. Moreover, some of the exercise variables are used as prognostic factors, mainly in pulmonary arterial hypertension (PAH) and chronic thromboembolic pulmonary hypertension (CTEPH) [4, 5]. In CTEPH, CPET is useful in detecting the disease, establishing severity, and identifying causes of exercise intolerance [6, 7]. Likewise, it has been described that patients with CTEPH have greater dead space, ventilatory inefficiency, and more severe alterations in gas exchange during exercise than patients with PAH [8].

High altitude is an elevation over 2500 m (~8200 feet) [9]. Although the physiological responses to hypobaric hypoxia start at lower elevations, they are more pronounced above this altitude, and the risk of developing altitude illness also increases substantially [10]. At altitude, the barometric pressure (BP) decreases, meaning the inspired oxygen pressure (${\mathrm{PIO}}_{2}$) and arterial oxygen pressure (${\mathrm{PaO}}_{2}$) also decrease. In Bogotá, a city located at high altitude (2640 m, BP: 560 mmHg), the ${\mathrm{PaO}}_{2}$ at rest in healthy subjects is around 65 mmHg, with values lower than 60 mmHg in older individuals [11]; values are even lower in patients with cardiopulmonary disease. In patients with chronic obstructive pulmonary disease (COPD) living at high altitude, we have observed more severe gas exchange alterations during exercise than described at sea level, mainly in those with a higher degree of obstruction or with coexistent PH [12, 13].

In patients with PH who live at high altitude, in addition to the pathophysiological alterations related to pulmonary vascular compromise, changes related to the decrease in ${\mathrm{PIO}}_{2}$ are added, meaning gas exchange alterations in these patients are expected to be more severe than those described at sea level. Since there are no studies evaluating the impact of high altitude on exercise capacity, ventilatory and cardiovascular responses, and gas exchange in patients with PH, we designed this study to describe and compare these variables in a CPET between patients with PAH and CTEPH.

## 2. Materials and Methods

### 2.1 Subjects

A retrospective study was performed using 53 consecutive patients with PH referred from the institution’s pulmonary vascular disease program between 2015 and 2020 to the Pulmonary Function Tests Laboratory of the Fundacion Neumologica Colombiana in Bogotá, Colombia (2640 m) for a CPET. The Institution’s Research Ethics Committee approved the conduct of the study and the anonymous use of the data (authorization number: 202111-26803). A control group of 102 subjects of similar age and with normal spirometry was used to reference the normal response during exercise at altitude. The control subjects were required to have no history of cardiopulmonary disease, obesity, or smoking.

To exclude secondary changes to the ascent to altitude, all patients and controls were to have been born and currently reside in Bogotá. Patients with PH should have been clinically stable for at least 6 weeks and without changes in targeted treatment for PH in the last 2 months. New York Health Association (NYHA) functional classification data and medications were recorded at the time of CPET. Hemodynamic variables were obtained from resting right heart catheterization (RHC) performed in the last three months.

### 2.2 PH Definitions

PAH was defined as a mean pulmonary arterial pressure (PAPm) ≥25 mmHg with a pulmonary artery wedge pressure (PAWP) ≤15 mmHg and a PVR >3 Wood units (WU) assessed in an RHC, in the absence of other causes of PH, such as PH due to lung diseases, CTEPH, or other rare diseases [1, 14]. Patients with idiopathic PH or PH associated with connective tissue disease were included in the PAH group. CTEPH was defined as mPAP ≥25 mmHg with PAWP ≤15 mmHg and at least one perfusion defect detected by lung scanning, multidetector computed tomographic angiography, or pulmonary angiography after ≥3 months of effective anticoagulation [1, 15].

### 2.3 Functional Tests at Rest

Spirometry and diffusing capacity of the lung for carbon monoxide (DLCO) were performed on a V-MAX Encore (CareFusion, Yorba Linda, CA, USA) according to the standards of the American Thoracic Society and European Respiratory Society and Crapo reference equations were used [16, 17, 18]. A certified 3 L syringe was used for calibration. Flows and volumes were reported according to BTPS conditions (body temperature, ambient pressure, saturated with water vapor).

### 2.4 Exercise Test

All patients performed a symptom-limited incremental test on a cycle ergometer that began with a 3-minute rest period, followed by 3 minutes of unloaded pedaling, and a subsequent increase in workload every minute until the maximum tolerated level was reached [19]. The increment (10–25 watts) was selected depending on the reported exercise tolerance and resting functional impairment. The work rate (WR), oxygen uptake (${\mathrm{VO}}_{2}$), ${\mathrm{CO}}_{2}$ production (${\mathrm{VCO}}_{2}$), minute ventilation (VE), tidal volume (VT), respiratory frequency (fR), heart rate (HR), oxygen pulse (${\mathrm{VO}}_{2}$/HR), end-tidal carbon dioxide tension (${\mathrm{PETCO}}_{2}$), and VE/${\mathrm{VCO}}_{2}$ were recorded as mean values for 30 s throughout the test. For data analysis, the average of these variables was evaluated during 3 min of rest and in the last minute of peak exercise. ${\mathrm{VO}}_{2}$ values were compared with the reference values ​​of Hansen *et al*. [20] and Wasserman *et al*. [21].

The arterial blood gases (ABG) sample was taken at rest and peak exercise. The alveolar–arterial oxygen tension gradient (${\mathrm{PA-aO}}_{2}$) was calculated using the alveolar gas equation: ${\mathrm{FIO}}_{2}$ × (BP – 47) – carbon dioxide arterial pressure (${\mathrm{PaCO}}_{2}$) × [${\mathrm{FIO}}_{2}$ + {1 – ${\mathrm{FIO}}_{2}$}/RER] – ${\mathrm{PaO}}_{2}$, where ${\mathrm{FIO}}_{2}$ (inspired fraction of oxygen) = 0.2093, mean BP = ~560 mmHg, and RER = measured respiratory exchange ratio. To evaluate changes with exercise, the delta (peak exercise – rest) of ${\mathrm{PaO}}_{2}$ and ${\mathrm{PaCO}}_{2}$ was calculated in controls and patients with PH. Using the ${\mathrm{PaCO}}_{2}$ and ${\mathrm{PETCO}}_{2,}$ the dead space to tidal volume ratio (VD/VT) was estimated. The anaerobic threshold (AT) was determined using the V-slope method [19]. Dyspnea and muscle fatigue were assessed using the Borg scale [22].

### 2.5 Data Analysis

Continuous variables are presented as the mean and standard deviation or median and interquartile range according to their distribution following evaluation by the Kolmogorov–Smirnov test. Qualitative variables are presented as proportions. To compare the variables at rest and peak exercise between the 3 groups (PAH, CTEPH, and controls), the nonparametric Kruskal–Wallis test or the one-way ANOVA test was used, with the Bonferroni post hoc test applied for multiple comparisons. The $X^{2}$ test was used to compare proportions. The change between rest and peak exercise (delta) of ${\mathrm{PaO}}_{2}$ and ${\mathrm{PaCO}}_{2}$ in each group was evaluated using the paired *t*-test. The SPSS (IBM SPSS Statistics, Version 22.0, Armonk, NY, USA) was used for data analysis, and a *p* < 0.05 was considered significant.

## 3. Results

### 3.1 Patients and Controls Characteristics

A total of 53 patients with PH were analyzed: 73.6% women, 29 in the PAH group, and 24 in the CTEPH group. The 102 controls included were the same age, sex, and body mass index (BMI) as the PH patients. Patients with PAH were younger (*p* < 0.05) and with a lower NYHA class (*p* = 0.037) compared with the CTEPH group. There were no differences between PAH and CTEPH in sex, BMI, hemoglobin, smoking history (13.8 vs. 20.8; *p* = 0.715), pack-years (4.1 ± 4.6 vs. 5.8 ± 2.8; *p* = 0.578), DLCO or hemodynamic variables (Table 1). The forced expiratory volume in the first second (${\mathrm{FEV}}_{1}$)/forced vital capacity (FVC) ratio was lower in CTEPH than in PAH (*p* = 0.005). Compared to CTEPH, a higher percentage of patients with PAH were on phosphodiesterase type 5 inhibitors (27.6% vs. 4.2%, *p* = 0.031), and none were on cyclic guanosine monophosphate stimulators (0.0% vs. 20.8%, *p* = 0.015). There were no differences between patients with PAH and CTEPH in the use of endothelin receptor antagonists (*p* = 0.242) or prostanoids (*p* = 0.649).

**Table 1.** **Clinical characteristics, lung function and hemodynamic variables**.

Variable		Controls	PAH	CTEPH	*p*
		N = 102	N = 29	N = 24	
Age, years		50.0 ± 14.3	45.2 ± 13.4	55.5 ± ${\mathrm{14.5}}^{b}$	0.034
Women		71 (69.6)	22 (75.9)	17 (70.8)	0.807
BMI, ${\mathrm{kg/m}}^{2}$		26.0 ± 3.3	25.3 ± 3.4	27.4 ± 4.2	0.099
Smoking history		-	4 (13.8)	5 (20.8)	0.715
Hb, gr/dL		15.2 ± 1.3	14.9 ± 2.3	15.4 ± 1.8	0.597
FVC, % predicted		104.9 ± 12.7	98.2 ± ${\mathrm{14.8}}^{a}$	92.8 ± ${\mathrm{17.4}}^{a}$	<0.001
${\mathrm{FEV}}_{1}$, % predicted		103.2 ± 13.1	92.6 ± ${\mathrm{16.0}}^{a}$	83.7 ± ${\mathrm{15.5}}^{a}$	<0.001
${\mathrm{FEV}}_{1}$/FVC, %		80.6 ± 5.3	78.4 ± 5.8	73.4 ± ${\mathrm{6.8}}^{a,b}$	<0.001
DLCO, % predicted		-	86.6 ± 21.1	77.2 ± 17.9	0.146
NYHA ≥2		-	18 (62.1)	21 (87.5)	0.037
Hemodynamics		-			
	∙ PAPm, mmHg		36.0 (28.5–62.0)	45.0 (35.0–56.0)	0.454
	∙ PVR, WU		6.2 (3.6–15.0)	6.9 (5.5–10.9)	0.628
	∙ PAWP, mmHg		12.0 (11.0–14.0)	13.0 (11.0–17.0)	0.055
	∙ RAP, mmHg		10.0 (7.5–13.0)	12.5 (10.0–14.0)	0.054
	∙ CI, L/min/$m^{2}$		2.9 (2.5–3.5)	2.7 (2.4–3.3)	0.685
	∙ ${\mathrm{SvO}}_{2}$, %		67.0 (65.0–71.0)	66.0 (62.5–72.0)	0.415

Values as a mean ± SD, median ($P_{25-75}$) or N (%). BMI, body mass index; CI, cardiac index; CTEPH, chronic thromboembolic pulmonary hypertension; DLCO, carbon monoxide diffusion capacity; ${\mathrm{FEV}}_{1}$, forced expiratory volume in the first second; FVC, forced vital capacity; Hb, hemoglobin; NYHA, New York Heart Association functional class; PAH, pulmonary arterial hypertension; PAPm, mean pulmonary artery pressure; PAWP, pulmonary artery wedge pressure; PVR, pulmonary vascular resistance; RAP, right atrial pressure; ${\mathrm{SvO}}_{2}$, mixed venous oxygen saturation; WU, Wood units. *p*: one-way ANOVA, Kruskal–Wallis or $X^{2}$. ^a^*p* < 0.05 vs. controls. ^b^*p* < 0.05 PAH vs. CTEPH.

### 3.2 Exercise Capacity and Cardiovascular Response

**Fig. 1.**
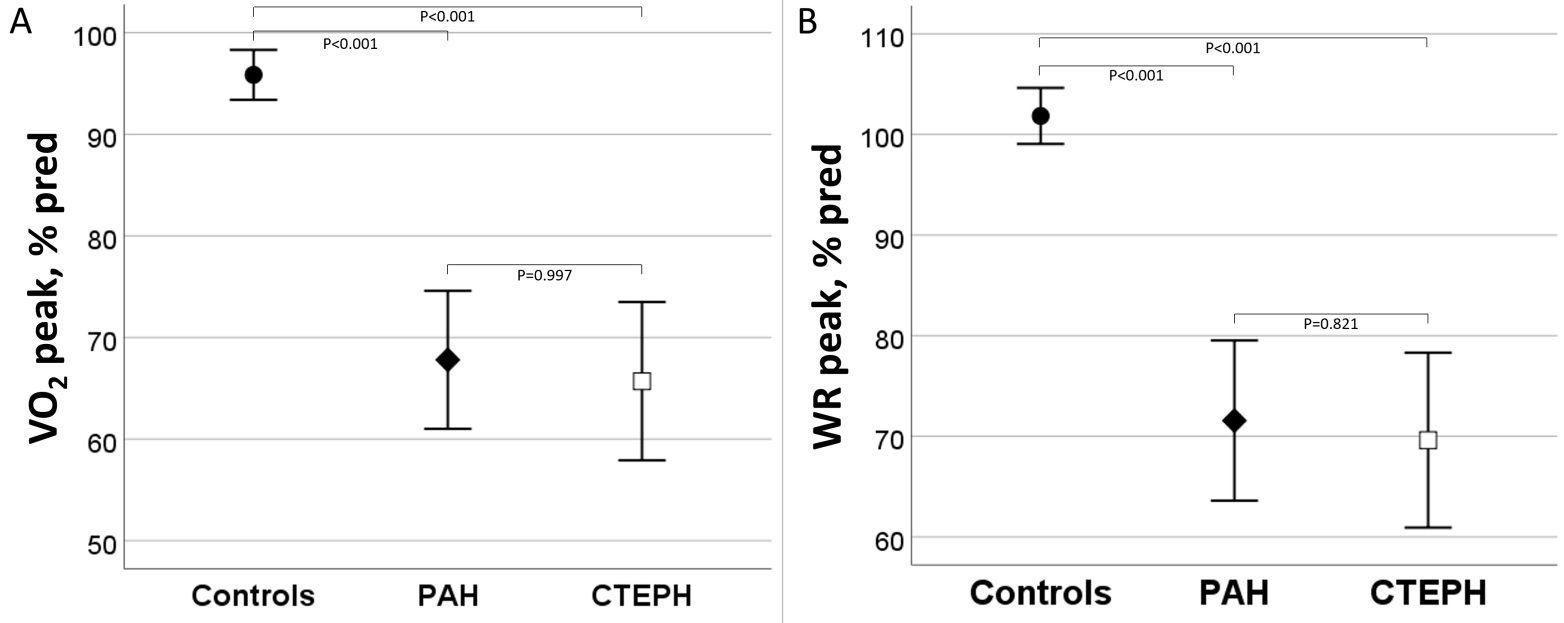
**Oxygen consumption and work rate at peak exercise in controls, PAH and CTEPH.** (A) Oxygen consumption (${\mathrm{VO}}_{2}$) and (B) work rate (WR) in peak exercise were significantly lower in PAH and CTEPH than in controls, with no differences between PAH and CTEPH. PAH, pulmonary arterial hypertension; CTEPH, chronic thromboembolic pulmonary hypertension.

At peak exercise, patients with PH had a lower ${\mathrm{VO}}_{2}$, WR, and ${\mathrm{VO}}_{2}$/HR than controls (*p* < 0.001). There were no differences in ${\mathrm{VO}}_{2}$% predicted (67.8 ± 18.7 vs. 66.0 ± 19.8), WR% predicted (71.9 ± 21.4 vs. 69.8 ± 21.5), and ${\mathrm{VO}}_{2}$/HR (88.0 ± 24.3 vs. 86.8 ± 29.8) at peak exercise between patients with PAH and CTEPH (Table 2) (Fig. 1).

**Table 2.** **Peak exercise variables in controls and PAH and CTEPH patients**.

Variable	Controls	PAH	CTEPH	*p*
	N = 102	N = 29	N = 24	
WR, % predicted	101.8 ± 14.2	71.9 ± ${\mathrm{21.4}}^{a}$	69.8 ± ${\mathrm{21.5}}^{a}$	<0.001
Peak ${\mathrm{VO}}_{2}$, % predicted	95.8 ± 12.5	67.8 ± ${\mathrm{18.7}}^{a}$	66.0 ± ${\mathrm{19.8}}^{a}$	<0.001
${\mathrm{VO}}_{2}$/kg peak, mL/kg per min	25.4 ± 6.7	18.1 ± ${\mathrm{4.3}}^{a}$	15.7 ± ${\mathrm{4.0}}^{a}$	<0.001
${\mathrm{VO}}_{2}$ AT, % predicted	57.6 ± 12.7	46.5 ± ${\mathrm{15.4}}^{a}$	48.3 ± ${\mathrm{16.7}}^{a}$	<0.001
${\mathrm{∆VO}}_{2}$/ΔWR, mL/min per W	10.8 ± 1.7	8.3 ± ${\mathrm{2.1}}^{a}$	8.3 ± ${\mathrm{2.0}}^{a}$	<0.001
RER	1.18 ± 0.08	1.16 ± 0.11	1.09 ± ${\mathrm{0.08}}^{a,b}$	<0.001
HR, % predicted	88.5 ± 6.0	76.6 ± ${\mathrm{12.1}}^{a}$	78.7 ± ${\mathrm{12.7}}^{a}$	<0.001
${\mathrm{VO}}_{2}$/HR, % predicted	108.7 ± 15.4	88.0 ± ${\mathrm{24.3}}^{a}$	86.8 ± ${\mathrm{29.8}}^{a}$	<0.001
VE, L/min	73.2 ± 20.9	54.2 ± ${\mathrm{16.6}}^{a}$	56.3 ± ${\mathrm{18.5}}^{a}$	<0.001
VT, mL/min	1842.9 ± 531.6	1431.9 ± ${\mathrm{339.6}}^{a}$	1465.4 ± ${\mathrm{649.4}}^{a}$	<0.001
fR, rpm	39.8 ± 7.6	37.7 ± 7.3	40.5 ± 7.3	0.340
VE/MVV, %	59.3 ± 10.7	48.3 ± ${\mathrm{12.4}}^{a}$	57.5 ± ${\mathrm{12.8}}^{a,b}$	<0.001
VE/${\mathrm{VCO}}_{2}$ nadir	34.2 ± 3.5	39.3 ± ${\mathrm{5.6}}^{a}$	45.8 ± ${\mathrm{7.1}}^{a,b}$	<0.001
Leg discomfort, Borg	5.8 ± 2.8	6.2 ± 2.6	5.4 ± 2.0	0.558
Dyspnea, Borg	5.1 ± 2.3	4.3 ± 2.0	6.1 ± ${\mathrm{2.7}}^{b}$	0.020
Dyspnea/VE peak	0.08 ± 0.04	0.09 ± 0.05	0.13 ± ${\mathrm{0.09}}^{a,b}$	<0.001

Values as a mean ± SD. AT, anaerobic threshold; CTEPH, chronic thromboembolic pulmonary hypertension; fR, respiratory rate; HR, heart rate; MVV, maximum voluntary ventilation; PAH, pulmonary arterial hypertension; RER, respiratory exchange ratio; VE/${\mathrm{VCO}}_{2}$, respiratory equivalent of ${\mathrm{CO}}_{2}$; VE, minute ventilation; VT, tidal volume; ${\mathrm{VO}}_{2}$, oxygen consumption; WR, work rate; W, mL/min per watt. *p*: one-way analysis of variance (ANOVA), Kruskal–Wallis or $X^{2}$. ^a^*p* < 0.05 vs. controls. ^b^*p* < 0.05 PAH vs. CTEPH.

### 3.3 Ventilatory and Gas Exchange Response

During exercise, control subjects achieved a higher VE and VT and lower VE/${\mathrm{VCO}}_{2}$ than patients with PH (*p* < 0.001). Further, PH patients had a higher VD/VT and ${\mathrm{PA-aO}}_{2}$ and lower ${\mathrm{PaO}}_{2}$ and ${\mathrm{SaO}}_{2}$ at rest and peak exercise than controls.

**Fig. 2.**
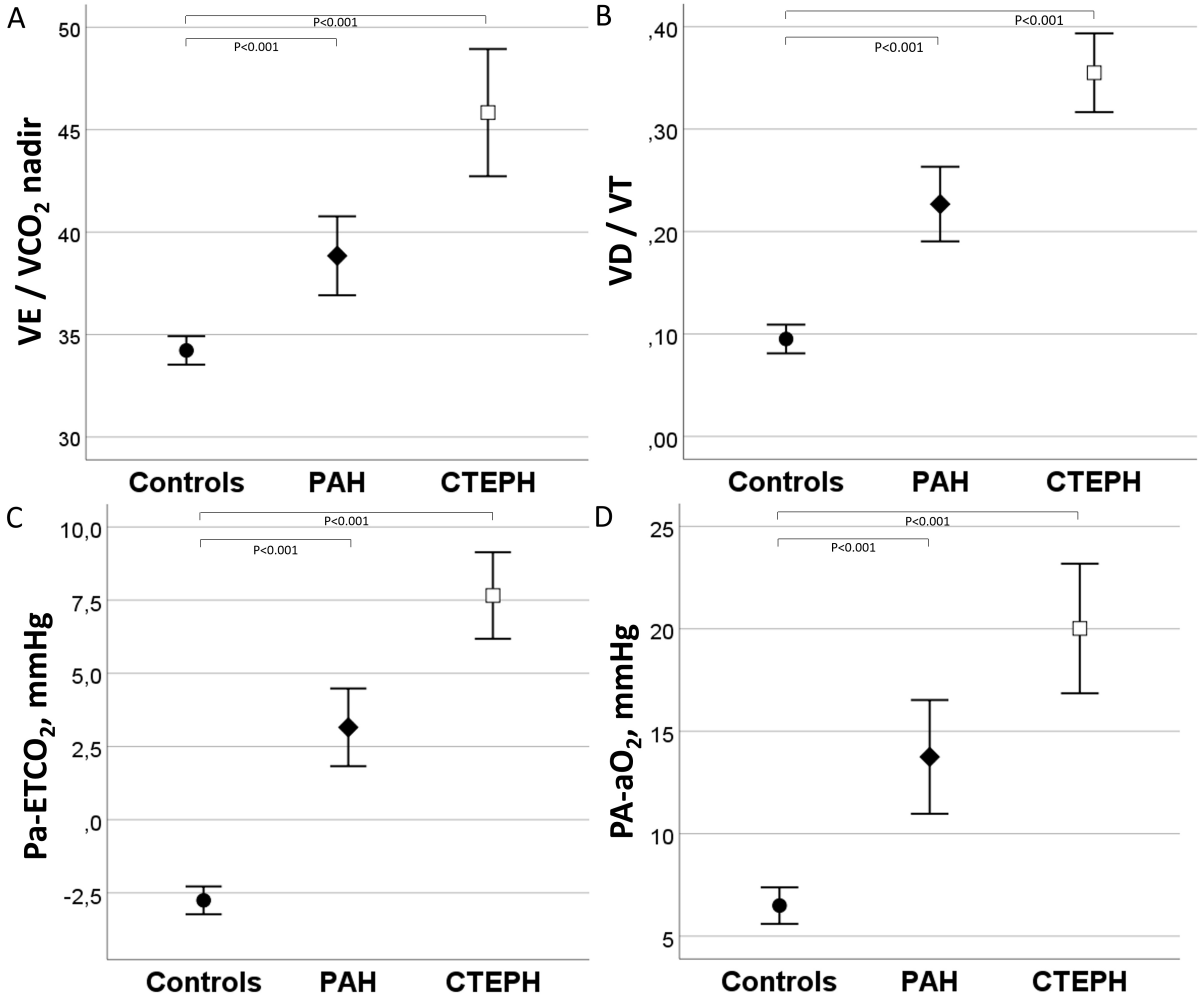
**VE/${\mathrm{VCO}}_{2}$, VD/VT, Pa-${\mathrm{ETCO}}_{2}$, and ${\mathrm{PA-aO}}_{2}$ during exercise in controls and PAH and CTEPH patients.** The (A) VE/${\mathrm{VCO}}_{2}$, (B) VD/VT, (C) Pa-${\mathrm{ETCO}}_{2}$, and (D) ${\mathrm{PA-aO}}_{2}$ were significantly higher in patients with CTEPH than PAH. CTEPH, chronic thromboembolic pulmonary hypertension; PAH, pulmonary arterial hypertension; VE/${\mathrm{VCO}}_{2}$, respiratory equivalent of ${\mathrm{CO}}_{2}$; VD/VT, dead space to tidal volume ratio; ${\mathrm{PA-aO}}_{2}$, alveolar–arterial oxygen pressure gradient; Pa-${\mathrm{ETCO}}_{2}$, arterial–ET carbon dioxide pressure gradient.

There were no differences in VE, VT, and fR at peak exercise between the PAH and CTEPH groups. The VE/MVV was higher in CTEPH than PAH (Table 2). At rest, the CTEPH patients had a higher VD/VT and ${\mathrm{PA-aO}}_{2}$ and lower ${\mathrm{PaO}}_{2}$ and ${\mathrm{SaO}}_{2}$ than PAH patients. Additionally, during exercise, patients with CTEPH had higher VD/VT (0.36 ± 0.09 vs. 0.23 ± 0.9, *p* < 0.001), VE/${\mathrm{VCO}}_{2}$ (45.8 ± 7.1 vs. 39.3 ± 5.6, *p* < 0.001), and ${\mathrm{PA-aO}}_{2}$ (19.9 ± 7.6 vs. 13.5 ± 7.6, *p* < 0.001), and lower ${\mathrm{PaO}}_{2}$ (58.3 ± 8.7 vs. 67.4 ± 8.7, *p* < 0.001) and ${\mathrm{SaO}}_{2}$ (87.8 ± 4.4 vs. 91.2 ± 4.3, *p* < 0.001) than those with PAH (Fig. 2). There were no differences between groups in ${\mathrm{PaCO}}_{2}$ values at rest and during peak exercise (*p* = 0.107). The ${\mathrm{PaO}}_{2}$ delta during exercise in the controls was 11.2 ± 5.3 mmHg (*p* < 0.001); in the patients with PAH, it was 4.3 ± 7.1 mmHg (*p* = 0.004), and 0.6 ± 6.1 mmHg (*p* = 0.670) in those with CTEPH. The ${\mathrm{PaCO}}_{2}$ delta in the controls was –2.9 ± 2.7 mmHg (*p* < 0.001); in the patients with PAH, it was –0.8 ± 3.3 mmHg (*p* = 0.185), and 0.2 ± 2.6 mmHg (*p* = 0.684) in those with CTEPH. The Pa-${\mathrm{ETCO}}_{2}$ gradient at peak exercise was significantly higher in PH patients compared with healthy controls but also was significantly superior in CTEPH than PAH (7.4 ± 3.5 vs. 2.9 ± 3.6, *p* < 0.001) (Table 3) (Fig. 2).

**Table 3.** **ABG at rest and peak exercise in controls and PAH and CTEPH patients**.

Variable	Rest				Peak exercise			
	Control	PAH	CTEPH	*p*	Control	PAH	CTEPH	*p*
Subjects	102	29	24		102	29	24	
${\mathrm{PaCO}}_{2}$, mmHg	31.0 ± 2.3	32.0 ± ${\mathrm{3.0}}^{a}$	31.8 ± 3.5	0.107	28.1 ± 2.8	31.0 ± ${\mathrm{4.1}}^{a}$	32.2 ± ${\mathrm{3.3}}^{a}$	<0.001
${\mathrm{PaO}}_{2}$, mmHg	66.6 ± 4.9	62.7 ± ${\mathrm{5.5}}^{a}$	57.7 ± ${\mathrm{7.5}}^{a,b}$	<0.001	77.8 ± 5.5	67.4 ± ${\mathrm{8.7}}^{a}$	58.3 ± ${\mathrm{8.7}}^{a,b}$	<0.001
${\mathrm{SaO}}_{2}$, %	93.6 ± 1.8	91.9 ± ${\mathrm{2.6}}^{a}$	90.0 ± ${\mathrm{3.7}}^{a,b}$	<0.001	94.5 ± 1.7	91.2 ± ${\mathrm{4.3}}^{a}$	87.8 ± ${\mathrm{4.4}}^{a,b}$	<0.001
${\mathrm{PA-aO}}_{2}$, mmHg	6.4 ± 4.4	9.1 ± ${\mathrm{4.4}}^{a}$	14.4 ± ${\mathrm{7.6}}^{a,b}$	<0.001	6.5 ± 4.5	13.5 ± ${\mathrm{7.6}}^{a}$	19.9 ± ${\mathrm{7.6}}^{a,b}$	<0.001
${\mathrm{PETCO}}_{2}$, mmHg	29.7 ± 2.9	29.2 ± 4.0	25.8 ± ${\mathrm{3.6}}^{a,b}$	<0.001	30.9 ± 3.1	28.2 ± ${\mathrm{4.2}}^{a}$	24.7 ± ${\mathrm{3.7}}^{a,b}$	<0.001
VD/VT	0.29 ± 0.08	0.32 ± 0.07	0.41 ± ${\mathrm{0.06}}^{a,b}$	<0.001	0.10 ± 0.07	0.23 ± ${\mathrm{0.09}}^{a}$	0.36 ± ${\mathrm{0.09}}^{a,b}$	<0.001
Pa-${\mathrm{ETCO}}_{2}$, mmHg	1.2 ± 2.8	2.7 ± ${\mathrm{2.8}}^{a}$	6.0 ± ${\mathrm{3.6}}^{a,b}$	<0.001	–2.8 ± 2.4	2.9 ± ${\mathrm{3.6}}^{a}$	7.4 ± ${\mathrm{3.5}}^{a,b}$	<0.001

Values as a mean ± SD. CTEPH, chronic thromboembolic pulmonary hypertension; PAH, pulmonary arterial hypertension; ${\mathrm{PaO}}_{2}$, partial pressure of arterial oxygen; ${\mathrm{PA-aO}}_{2}$, alveolar–arterial oxygen pressure gradient; Pa-${\mathrm{ETCO}}_{2}$, arterial–ET carbon dioxide pressure gradient; ${\mathrm{PETCO}}_{2}$, carbon dioxide end-tidal pressure; ${\mathrm{SaO}}_{2}$, oxygen arterial saturation; VD/VT, dead space to tidal volume ratio; ABG, arterial blood gases; ${\mathrm{PaCO}}_{2}$, carbon dioxide arterial pressure. *p*: one-way analysis of variance (ANOVA). ^a^*p* < 0.05 vs. controls. ^b^*p* < 0.05 PAH vs. CTEPH.

### 3.4 Symptoms

At peak exercise, there was no difference in the fatigue of the lower limbs between PAH, CTEPH patients, and controls (*p* = 0.558). Analysis using the Borg scale revealed dyspnea (6.1 ± 2.7 vs. 4.3 ± 2.0, *p* = 0.016) and dyspnea/VE (0.13 ± 0.09 vs. 0.09 ± 0.05, *p* = 0.031) were higher in patients with CTEPH than PAH (Table 2).

## 4. Discussion

The main findings of this study, which assessed a significant number of PH patients residing at high altitude were the following: (1) In comparison to the controls, PH patients had lower exercise capacity (peak ${\mathrm{VO}}_{2}$ and WR) and severe gas exchange alterations. (2) There was no difference in exercise capacity between PAH and CTEPH patients. (3) There was more dyspnea, greater ventilatory inefficiency, and more severe gas exchange alterations during exercise in patients with CTEPH than with PAH. (4) Compared to what is described at sea level, due to the lower ${\mathrm{PIO}}_{2}$ at altitude, for both CTEPH and PAH, the ${\mathrm{PaO}}_{2}$ and ${\mathrm{SaO}}_{2}$ during exercise were lower, and due to the compensatory increase in ventilation, the VE/${\mathrm{VCO}}_{2}$ ratio was greater.

In this study, in subjects living at high altitude, as expected, there was lower exercise capacity (peak ${\mathrm{VO}}_{2}$ and WR) and severe alterations in gas exchange in patients with PH compared to controls. PAH and CTEPH patients had lower ${\mathrm{VO}}_{2}$/HR and higher VE/${\mathrm{VCO}}_{2}$, VD/VT, Pa-${\mathrm{ETCO}}_{2}$, hypoxemia, and ${\mathrm{PA-aO}}_{2}$ than controls, which is related to pulmonary vascular compromise [4, 23]. These lower ${\mathrm{VO}}_{2}$/HR values manifest the alteration in the stroke volume that can be seen in the presence of PH. The high VE/${\mathrm{VCO}}_{2}$ ratio is a hallmark abnormality in patients with pulmonary vascular disease, primarily resulting from high VD/VT. In patients with PH, an increase in the VE/${\mathrm{VCO}}_{2}$ ratio has been related to different, usually coexisting mechanisms, including high VD/VT, as already mentioned, abnormalities in gas exchange, increased chemosensitivity, and an abnormal ${\mathrm{PaCO}}_{2}$ set point [24]. The higher Pa-${\mathrm{ETCO}}_{2}$ and ${\mathrm{PA-aO}}_{2}$ values reflect ventilation/perfusion imbalance [4, 23, 24].

Although there were no differences between PAH and CTEPH in hemodynamics, peak ${\mathrm{VO}}_{2}$, and WR, similar to previous studies conducted at sea level [8, 25, 26], the ventilatory inefficiency and gas exchange alterations in CTEPH during exercise were more severe in comparison to PAH, with higher VE/${\mathrm{VCO}}_{2}$, VD/VT, Pa-${\mathrm{ETCO}}_{2}$, and ${\mathrm{PA-aO}}_{2}$, and lower ${\mathrm{PaO}}_{2}$ and ${\mathrm{SaO}}_{2}$. We highlight that, unlike controls, which significantly increased ${\mathrm{PaO}}_{2}$ during exercise, the increase in patients with PAH was much smaller, while there was no significant change in those with CTEPH. Similarly, in normal subjects, ${\mathrm{PaCO}}_{2}$ decreased significantly from exercise but did not change in those with PAH and CTEPH.

Consistent with our results, a previous study described significantly lower ${\mathrm{PETCO}}_{2}$ and significantly higher end-tidal capillary carbon dioxide gradients in CTEPH versus PAH, both at rest and during exercise. It has been described that a gradient >7.0 mmHg would indicate CTEPH with a sensitivity of 75% at rest and 88% during exercise, which suggests the usefulness of this variable in the differential diagnosis between these two pathologies [26].

Several potential mechanisms could explain the differences in gas exchange and ventilatory efficiency during exercise between PAH and CTEPH. In CTEPH, there is anatomical compromise and heterogeneity in pulmonary blood flow. In addition to intravascular obstruction of the pulmonary arteries by unresolved organized fibrotic clots, pulmonary vascular remodeling can lead to severe pulmonary microvasculopathy, which affects the small muscular pulmonary arteries, pulmonary capillaries, and veins. Enlargement and proliferation of systemic bronchial arteries also occur, as well as anastomoses between the systemic and pulmonary circulations that promote the development of microvasculopathy [27, 28].

Although most patients with PH were non-smokers and those who had smoked had a low pack-year index, the ${\mathrm{FEV}}_{1}$/FVC ratio was lower in patients with PH than in controls, mainly in those with CTEPH. Even though restrictive alteration in pulmonary function tests has been described in patients with PH [29], obstructive ventilatory alteration has also been reported in both PAH [29, 30, 31] and CTEPH [8, 26, 32, 33, 34]. Similar to our data, in some studies that compared spirometric values between patients with PH, the ${\mathrm{FEV}}_{1}$ and the ${\mathrm{FEV}}_{1}$/FVC ratio were lower in patients with CTEPH than in PAH [8, 26, 32].

The decrease in ${\mathrm{FEV}}_{1}$ or the ${\mathrm{FEV}}_{1}$/FVC ratio has been attributed to different possible mechanisms, such as the involvement of the peripheral airways, effects of vasoactive or inflammatory substances, compression of the airways related to arterial dilation, and, less likely, to inspiratory muscle dysfunction [29, 30, 34, 35].

Dyspnea also was more severe in CTEPH patients. Although the exact mechanisms related to dyspnea in PH patients are not completely understood [4], the increased dyspnea in the CTEPH group was probably explained by the higher VD/VT [8, 26] and ventilatory inefficiency and more severe gas exchange alterations than in PAH patients. Although we did not perform inspiratory capacity measurements through exercise, another possible mechanism that could be related to the increased exertional dyspnea intensity in these patients is dynamic hyperinflation (DH) [4, 35].

It is estimated that over 500 million humans live at ≥1500 m, 81.6 million at ≥2500 m, and 14.4 million at ≥3500 m [36]. Living at altitude imposes a challenge on humans due to changes in oxygen pressure and climatic variables. The reduction in BP and the consequent decrease in ${\mathrm{PIO}}_{2}$ in the atmosphere condition changes the ventilatory pattern and causes a decrease in oxygenation, which is more pronounced in patients with cardiopulmonary disease. In previous studies at the same altitude as Bogotá, we have shown, compared to studies at sea level, a high prevalence of PH in patients with COPD, particularly in patients with less severe airflow obstruction [37], and more PH in patients with idiopathic pulmonary fibrosis [38]. In these patients, the ${\mathrm{PaO}}_{2}$ at rest or during exercise is lower than that reported at sea level [12, 38] and even lower in patients with COPD and the coexistence of PH [13]. Although there are several pathophysiological mechanisms related to the development of PH, alveolar hypoxia at high altitudes is probably a fundamental factor in promoting and developing PH [39, 40]. Although hypoxia-inducible factor (HIF) signaling is a mechanism related to disease progression in group 3 PH (associated with lung diseases and/or hypoxia), increased HIF-1α has also been observed in the lung tissue of patients with PAH and CTEPH [41, 42].

In control subjects, ${\mathrm{PaCO}}_{2}$ and ${\mathrm{PETCO}}_{2}$ were lower and VE/${\mathrm{VCO}}_{2}$ higher compared to descriptions in normal subjects at sea level, which is explained by increased ventilation, a well-recognized compensatory mechanism for adaptation to altitude [43, 44]. Similarly, in PH patients residing at 2640 m, the VE/${\mathrm{VCO}}_{2}$ ratio was lower than the values described in various studies at sea level: 47 to 68 in patients with CTEPH [8, 25, 45, 46] and 42 to 54 in those with PAH [8, 25, 46, 47, 48, 49, 50, 51]. Regarding oxygenation, studies at sea level in patients with CTEPH have described values of ${\mathrm{PaO}}_{2}$ in exercise between 62 and 70 mmHg [8, 46] and for arterial oxygen saturation by pulse oximetry (${\mathrm{SpO}}_{2}$) between 91 and 93% [7, 8], which are higher than those found in our study. Similarly, in PAH patients, the ${\mathrm{PaO}}_{2}$ described during exercise at sea level was between 78 and 83 mmHg [8, 46], while ${\mathrm{SpO}}_{2}$ was between 89 and 92% [8, 48, 49], also higher than in these patients at high altitude. The lower ${\mathrm{PaO}}_{2}$ in subjects residing at high altitude can be explained, in addition to ventilation/perfusion ratio (V/Q) alterations related to the disease, by low ${\mathrm{PIO}}_{2}$ secondary to decreased BP [52].

In a recent study in patients with PAH and CTEPH, acute altitude exposure after ascending from 470 m to 2500 m caused a significant decrease in exercise capacity, ventilatory efficiency, and oxygenation [53]. It is striking that, despite these being patients at a slightly lower altitude than in our study, who also were in a better functional class and had lower pulmonary vascular resistance than the patients in our study, the level of hypoxemia at rest and during exercise was significantly higher. This indicates the presence of adaptive mechanisms in high-altitude resident subjects who are chronically exposed to hypoxia, which has been previously described in healthy subjects and in patients with other diseases, such as COPD [11, 12, 54].

This is the first study to evaluate exercise capacity and gas exchange alterations in patients with PH living at high altitude. Moreover, including patients with PAH, CTEPH, and control subjects allowed us to compare groups; meanwhile, measuring the ABG and ventilatory variables comprehensively assessed the limiting mechanisms of exercise in these patients with PH. Consistent with several studies at sea level, conducting this study at high altitude we also show a greater compromise in ventilatory efficiency and oxygenation in CTEPH than in PAH patients, with no differences in ${\mathrm{VO}}_{2}$ or peak WR between these groups [8, 25, 26]. However, unlike these studies, we observed modifications to the ventilatory pattern secondary to adaptive compensatory hyperventilation at altitude and lower oxygenation values, both in PAH and CTEPH [7, 8, 46, 48, 49], which can be explained by the lower ${\mathrm{PIO}}_{2}$ secondary to decreased BP [52].

We consider that the clinical utility of CPET is to evaluate exercise capacity in individual patients and identify alterations in gas exchange related to the pathophysiology of PAH and CTEPH that could explain the functional class and dyspnea of these patients. Considering that adaptive mechanisms are performed when living at different altitudes above sea level, we think these research data mainly apply to patients with PH who reside at high altitudes.

This study had several limitations, such as the retrospective design and the small sample size. Despite this, the patients in each group had a full evaluation and confirmation of the diagnosis at the institution’s pulmonary vascular disease group board using accepted diagnostic criteria. Even though DH has been linked to exertional dyspnea in some patients with PH [4, 35], we did not have inspiratory capacity and dyspnea measurements throughout the exercise to assess these dynamic changes.

Although at sea level, it has been established that there is a relationship between mortality in PH and some variables measured in CPET, such as peak ${\mathrm{VO}}_{2}$ and respiratory equivalents [4, 5], the results of these studies cannot be applied to patients who reside at high altitude due to the differences in the response to exercise, ventilatory efficiency and gas exchange variables related to the decrease in PB. For this reason, prospective studies in patients with PH are required to establish which physiological variables during exercise are related to mortality and which cut-off point has the best prognostic significance.

## 5. Conclusions

At high altitude, patients with PH present severe gas exchange alterations during exercise. Although there were no differences in hemodynamics at rest or in exercise capacity between patients with PAH and CTEPH, those with CTEPH had greater dyspnea, ventilatory inefficiency, and alterations in gas exchange during exercise. The CPET allowed the identification of these alterations related to the pathophysiology of the CTEPH that could explain the lower functional class and dyspnea in these patients.

## Data Availability

The data set used for our analysis is available upon request from the corresponding author.

## Abbreviations

AT, anaerobic threshold; BP, barometric pressure; CPET, cardiopulmonary exercise test; CTEPH, chronic thromboembolic pulmonary hypertension; ${\mathrm{FEV}}_{1}$, forced expiratory volume in 1 s; fR, respiratory frequency; FVC, forced vital capacity; HR, heart rate; MVV, maximal voluntary ventilation; ${\mathrm{PaCO}}_{2}$, carbon dioxide arterial pressure; Pa-${\mathrm{ETCO}}_{2}$, arterial end-tidal carbon dioxide pressure gradient; ${\mathrm{PaO}}_{2}$, oxygen arterial pressure; ${\mathrm{PA-aO}}_{2}$, alveolar–arterial oxygen gradient; PAH, pulmonary arterial hypertension; ${\mathrm{PETCO}}_{2}$, end-tidal carbon dioxide pressure; PH, pulmonary hypertension; ${\mathrm{PIO}}_{2}$, inspired oxygen pressure; RER, respiratory exchange ratio; ${\mathrm{SaO}}_{2}$, arterial oxygen saturation; ${\mathrm{VCO}}_{2}$, carbon dioxide production; ${\mathrm{VO}}_{2}$, oxygen uptake; ${\mathrm{VO}}_{2}$/HR, oxygen pulse; VD/VT, dead space to tidal volume ratio; VE/${\mathrm{VCO}}_{2}$, ventilatory equivalent for carbon dioxide; VE, minute ventilation; VT, tidal volume; WR, work rate.
